# Antimicrobial, Cytotoxic, and Anti-Inflammatory Activities of* Pimenta dioica* and* Rosmarinus officinalis* Essential Oils

**DOI:** 10.1155/2019/1639726

**Published:** 2019-05-07

**Authors:** Ana Cecilia Lorenzo-Leal, Enrique Palou, Aurelio López-Malo, Horacio Bach

**Affiliations:** ^1^Department of Medicine, Division of Infectious Diseases, University of British Columbia, Vancouver, BC, Canada; ^2^Chemical and Food Engineering Department, Universidad de las Americas Puebla, San Andres Cholula, Puebla 72810, Mexico

## Abstract

Essential oils (EOs) are natural products composed of a mixture of volatile and aromatic compounds extracted from different parts of plants that have shown antimicrobial activities against pathogens. In this study, EOs extracted from* Pimenta dioica *(Myrtaceae) and* Rosmarinus officinalis* (Lamiaceae) were assessed for their antimicrobial activities using a panel of pathogenic Gram-positive, Gram-negative, and fungal strains. The antimicrobial activity was measured by the minimal inhibitory concentration required for the growth inhibition of the microorganisms. The cytotoxicity of the EOs was tested* ex vivo* using the model of human-derived macrophage THP-1 cells. In addition, an inflammatory response was evaluated using the anti-inflammatory cytokine IL-10 and the proinflammatory cytokines IL-6 and TNF-*α*. Results showed that both EOs had antimicrobial activity and different pathogens were exposed to concentrations ranging between 600 and 2000 *μ*g/mL. In addition, the EOs showed no inflammatory activity when exposed to human macrophages, but a potent anti-inflammatory activity was measured when the oil from* Rosmarinus officinalis* was exposed to macrophages. This study demonstrates that the use of EOs is an effective alternative for pathogenic bacterial and fungal control, alone or in combination with antibiotic therapy. Moreover, the oil extracted from* Rosmarinus officinalis* could be used as potent anti-inflammatory agent.

## 1. Introduction 

Antibiotics are molecules used to treat infectious diseases. The appearance of multidrug-resistant strains of pathogens has alerted the scientific community and health care systems worldwide because of the lack of treatment for microbial-related illnesses [1, 2]. This threat has also been increased because of the misuse of antibiotics [3].

Natural products have been used in traditional medicine to treat infectious diseases since ancient times. Over the last few decades, the antimicrobial activity of these products has been scientifically validated [4].

Essential oils (EOs) are a mixture of volatile and aromatic compounds extracted from different parts of plants. EOs extracted from plants such as basil, cilantro, eucalyptus, and oregano have shown antimicrobial activities [5–7], including their potential to protect foods against pathogenic microorganisms [4, 8, 9].

Leaves of the tree* Pimenta dioica *(PD) (Myrtaceae) are used as ingredients in many cuisines worldwide. In addition, it has been used in traditional medicine to treat different illnesses [10–12].* Rosmarinus officinalis* (RO) (Lamiaceae) is an herb used worldwide in cuisine, and it can also be used in traditional medicine for its antimicrobial, antiparasitic, and antinociceptive activities; also, it is a strong candidate as an anti-inflammatory and a wound-healing agent [13–18].

Several compounds extracted from EOs have been reported to have antimicrobial activity. For example, citronellol, estragole, eudesmol, eugenol, geraniol, linoleic acid, and phytol have all shown significant antimicrobial activities against human and plant pathogens [19–24].

Following our program of investigation with the purpose of exploring new alternatives for antimicrobial activities based on EOs, we evaluated the antimicrobial activities of the EOs extracted from allspice (PD), and rosemary (RO) against a panel of pathogenic bacteria and fungi. The bacterial strains included Gram-positive and Gram-negative species, and the fungal strains included filamentous and yeast species. In addition, the cytotoxic and inflammatory activities of the EOs were assessed with a human macrophage cell line.

## 2. Experimental Section

### 2.1. EOs and Plant Material

RO EO was obtained from Hersol® laboratories (San Mateo Atenco, Estado de México, Mexico). Dried berries of PD were purchased from Condimentos Naturales Tres Villas S.A. de C.V. Puebla, Mexico.

### 2.2. PD EO Extraction and Sample Preparation

The dried berries of PD were first ground (NutriBullet, Magic Bullet, USA) and sieved (number 20 mesh, 850 *μ*m). The EO was extracted using a microwave-assisted extraction (MAE) method after mixing the ground material with water at a ratio of 1:20 (w/v). The microwave (NEOS System equipment, Milestone, Shelton CT, USA) was operated at 800 W and 600 W for 30 min each. The extracted oil was placed in hermetically sealed amber vials to avoid any volatilization of the component. Stock solution of the two EOs at concentrations of 20 mg/mL DMSO was prepared and stored at 4°C until needed.

The chemical analysis of both EOs was analyzed by gas chromatography equipped with a mass spectrometer, as published [25]. The main components of the PD EO were eugenol (~90%) and *α*-terpineol (2%), and the main components of RO EO were *α*-pinene (27%), camphor (21%), and 1,8 cineole (~21%) [25].

### 2.3. Strains and Culture Media

The pathogenic bacterial strains assayed in this study were* Acinetobacter baumannii* (ATCC BAA-747),* Escherichia coli* (ATCC 25922),* Pseudomonas aeruginosa* (ATCC 14210), methicillin-resistant* Staphylococcus aureus* (MRSA) (ATCC 700698), and* Staphylococcus aureus* (ATCC 25923). The pathogenic fungal strains included the yeast* Candida albicans* (ATCC 10231) and* Cryptococcus neoformans* var.* grubii* (kindly provided by Dr. Karen Bartlett, University of British Columbia, BC, Canada). The filamentous fungi* Aspergillus fumigatus* (ATCC 1022) and* Trichophyton rubrum* (ATCC 18758) were also tested in this study. Bacterial stocks were maintained in Mueller-Hinton broth (Becton & Dickinson) supplemented with 1.5% agar (Becton & Dickinson) at 4°C. Bacterial strains were cultured in a shaker at 37°C with the same broth. Fungal strains were maintained in Sabouraud broth (Becton & Dickinson) supplemented with 1.5% agar and incubated at 28°C. In the case of the filamentous fungi, spores were harvested in 1 mL of Sabouraud broth containing 10% glycerol, aliquoted, and maintained at -20°C until further use [26].

### 2.4. Minimal Inhibitory Concentration Determination

The minimum inhibitory concentration (MIC) was defined as the minimum concentration at which no growth was observed (no turbidity observed in the well). MICs were determined by a microdilution assay using a 96-well plate, according to previous published protocols [27]. The EO concentrations of 20, 30, 40, 50, 100, and 200 *μ*g/mL were assayed in a final volume of 100 *μ*L/well. Bacterial strains were grown at 37°C overnight and their densities were adjusted to an optical density of 0.05 at 600 nm, while 5 *μ*L of a spore suspension (1x10^6^ spores/mL) was used as inoculum for fungal strains, which were incubated at 28°C for 48 h. Untreated cells and DMSO were used as negative controls, whereas amikacin and gentamicin (for bacteria), and amphotericin and terbinafine (for fungi) were used as positive controls. Experiments were performed in triplicate.

### 2.5. Cytotoxic Assay

The cytotoxicity of the EOs was performed using human-derived THP-1 monocytic cells (ATCC TIB-202), following published protocols [9]. Briefly, 5x10^4^ cells were dispensed per well in a 96-well plate with a final volume of 100 *μ*L. EOs were tested at final concentrations of 2000, 1000, 600, 200, 100, 50, and 10 *μ*g/mL. The detergent Tween-20 (10 *μ*L of a 10% solution) was used as a positive control, whereas untreated cells and DMSO were used as negative controls. The analysis of the EO toxicity was performed with MTT (3-(4,5-dimethylthiazol-2-yl)-2,5-diphenyltetrazolium bromide) following published protocols [9]. The half-maximal lethal concentration (LC_50_) was calculated by plotting the EO concentrations against the damaged cells. Experiments were performed in triplicate. Final concentrations of DMSO per well were always ≤ 1%.

### 2.6. Anti-Inflammatory Assay

The anti-inflammatory assay was performed as previously published using activated THP-1 cells at a final concentration of 7.5x10^4^ cells/well [9]. Cells treated with 1% DMSO served as negative control, whereas 100 ng/mL of lipopolysaccharide (LPS) from* E. coli *(Sigma-Aldrich) was used as a positive control. Experiments were carried out in triplicate and the final concentrations of DMSO per well were always ≤ 1%. EOs were tested at a final concentration of 7.5 *μ*g/mL, which was selected based on the survival of the cell in the cytotoxic experiments.

### 2.7. Statistical Analysis

A t-test was used for statistical analysis. The statistical analysis was performed with Prism 4 (GraphPad Software, Inc.). A *p*-value <0.05 was considered statistically significant.

## 3. Results and Discussion

### 3.1. Antimicrobial Activities

The EOs were tested against two panels of pathogenic bacteria and fungi. Results showed that the EO extracted from PD was the most effective to kill five strains, including* A. baumannii*, MRSA,* P. aeruginosa*,* S. aureus*, and the yeast* C. albicans,* with MICs ranging between 500 and 2000 *μ*g/mL (Table 1). The bacterial strain* E. coli* was resistant to the concentrations tested in this study.

Our study addresses the control of human pathogens that have developed antimicrobial resistance and have caused hospital outbreaks and healthcare-associated infections in recent years, such as* A. baumannii* [28]. In addition, the EO also showed antibacterial activity against MRSA and* P. aeruginosa*, which have been public health problems worldwide because of their resistance to commonly used antibiotics [29, 30].

A previous study from Oussalah et al. [31], related to the antibacterial activity of the EO of PD, reported that the EO extracted from leaves showed antibacterial activity against* E. coli*,* Listeria monocytogenes*,* S. aureus*, and* Salmonella *Typhimurium, with MICs ranging between 0.1% and 0.2% [31]. Although these results indicate that a higher activity was shown in that study, the methodology was based on mixing the EO in molten agar, whereas our experiment was based on dissolving the EO in DMSO with direct supplementation to the bacterial broth. In addition, the PD EO used in Oussalah's study may have different percentages of the major components (data not shown in that study) of the EO, compared to our study (as described in Materials and Methods). This chemotypic difference depends on the geographic location of the plants, the methodology used for the EO extraction, season of the year, and environmental conditions in the region, with profound effect on the bioactivity of the EOs [32].

Regarding the antifungal activity, the PD EO was able to inhibit the growth of* C*.* albicans, *a yeast resistant to antifungal drugs [33]. Another study reported that the antifungal activity of the PD EO tested against* Fusarium oxysporum, F. verticillioides, Penicillium expansum, P. brevicompactum, Aspergillus flavus*, and* A. fumigatus* at a mean value of 0.6 *μ*L/mL [34]. These results cannot be compared to our results because of the different technique and fungal strains used in that study.

In the case of RO, the EO was able to inhibit the growth of* A. baumannii *at concentrations of 500 *μ*g/mL but was unable to inhibit the growth of the rest of the bacterial strains tested (Table 1). Interestingly, other studies have reported antibacterial activities against* E. coli*,* P. aeruginosa*, and* S. aureus,* with variable MICs ranging between 0.3 mg/mL and 1.72 mg/mL [35–37], which include our MIC of 0.5 mg/mL for* A. baumannii*. The different chemotypes of the RO EOs used in the different studies may suggest the reason why no activities against* E. coli*,* P. aeruginosa*, and* S. aureus* were observed in our study with concentrations < 2 mg/mL.

In our study, the RO EO was able to inhibit the growth of* C*.* albicans* in a similar concentration as PD (Table 1). A few studies reported the activity of RO EO against this yeast with discrepancies. For example, although in our study a MIC of 0.6 mg/mL was measured, higher MICs ranging between 5 mg/mL and ~10 mg/mL (1%) were measured in other reports [38, 39]. Also, a very low MIC of 5.6 *μ*L/mL was measured in a different study [40], but it is noteworthy that this low MIC was expressed as MIC_80_ and not MIC_100_ as in our study. Again, different chemotype oils may be the cause of the large difference in the MICs. Another study reported antifungal activity of RO EO against* F. verticillioides* with a calculated MIC of 150 *μ*g/mL [41]. Again, our results are not comparable to this study because the strain used was not in our screening panel of fungi.

The composition of essential oils is correlated with their antimicrobial activity. Phenolic compounds are known to have a major antibacterial activity compared to other chemical groups. The chemical function of the component could also decrease the EO antimicrobial activity, since phenols are usually more effective than cinnamic aldehydes, followed by alcohols, aldehydes, ketones, ethers, and hydrocarbons [42]. As mentioned in the Materials and Methods section, a previous study from our group reported that the eugenol was the major compound (~90%) of the PD EO [25]. Eugenol is a phenolic compound with reported antimicrobial activities [4] and was likely responsible for the antimicrobial activity in our study. Previous studies in which phenolic groups were assessed against plaque formations in the oral cavity show that eugenol significantly reduces the number of the plaques, compared to the placebo group [43]. In addition, eugenol at concentrations of 1000 *μ*g/mL inhibited the growth of* Streptococcus oralis*, a known oral pathogen responsible for cavities and periodontal disease development [44, 45]. Moreover, eugenol was able to inhibit the growth of* S. typhi* at a final concentration of 0.0125% after 60 min exposure [46]. In this report, the mechanism of eugenol was reported to increase the bacterial membrane permeability of the pathogen [46], as reported in* E. coli* and* L. monocytogenes* [47]. Another study reported that the mechanisms of action were due to a leakage of K^+^ from the cytosol of* E. coli* and* S. aureus* [48]. Both mechanisms can be connected to a leakage of K^+^ from the cytoplasm, which produces a shrinking of the cell as a result of changes in the turgor tension.

Eugenol was also reported as an antifungal agent against different pathogenic fungi. For example, in an* in vivo* study, guinea pigs were infected with* Microsporum gypseum* and thereafter treated with 0.01-0.03% of eugenol mixed in petroleum jelly. This formulation was effective not only to control the infection with concentrations similar to the nystatin used as a positive control, but also to improve the skin lesions [49]. Other studies using* C. albicans* were also reported. For example, an* in vivo* study of candidiasis performed in immunosuppressed rats showed that a daily treatment of eugenol (24 mM) reduced ~96% the number of CFU after 4 days of treatment [50]. Moreover, a broad study including the exposure of 31 clinical isolates of* C*.* albicans* strains to eugenol revealed that an averaged MIC of 625 *μ*g/mL inhibited the growth of all the tested strains [51]. Interestingly, our study reported that the same pathogen was inhibited by similar concentrations of the PD EO, suggesting that eugenol (95%) is responsible for the antifungal activities.

In the case of RO, the major components of the EO were *α*-pinene, 1,8-cineole or eucalyptol, and camphor [25]. The antibacterial activity of *α*-pinene has been reported against* E. coli* and* S. aureus*. Although no activity was found against* E. coli* as reported in our study,* S. aureus* was inhibited at concentrations of 13.6 *μ*g/mL [52]. Moreover, the mechanism of toxicity of this compound against* C. albicans* is based on the rupture of the membranes and cell wall, and the impairment of the production of DNA, RNA, ergosterol, and polysaccharides involved in the construction of the cell wall [53].

The second most abundant compound in the EO is 1,8-cineole, or eucalyptol, which has been reported as an antimicrobial agent. For instance, antimicrobial activities against a panel of bacteria and fungi ranging between 8 and 64 mg/mL were reported [54]. Microorganisms in this panel included the microorganisms used in our study. It is noteworthy that these MICs are elevated compared to our study, but we used the EO that contains only a fraction of eucalyptol compared to the pure compound used in this study. Similarly, other studies reported MICs ranging between 2 and 23 mg/mL and 8 and 64 mg/mL using panels of microorganisms that also included the strains reported in our study [55, 56].

### 3.2. Cytotoxic and Inflammatory Activities

**Figure 1:**
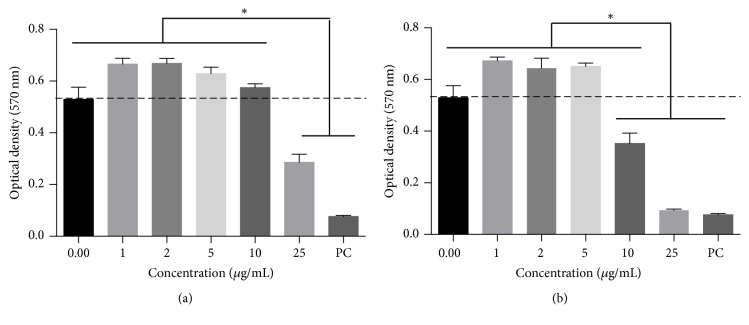
*Cytotoxicity of EO*. The cytotoxicity of the (a)* Pimenta dioica* and (b)* Rosmarinus officinalis* EOs was assessed on human-derived macrophage THP-1 cell line using the MTT assay. PC: positive control. Shown is the mean ± S.D. of three independent experiments. *∗*: *P*-value <0.05.

The cytotoxic and anti-inflammatory activities were assayed on the human macrophage cell line THP-1. When the cytotoxicity was assayed, the results showed that the EOs from PD and RO were toxic at concentration of 10 *μ*g/mL and 5 *μ*g/mL, respectively (Figures 1(a) and 1(b)). LD_50_ values of 29.63 *μ*g/mL and 14.15 *μ*g/mL were calculated for PD and RO EOs, respectively. A previous study performed in Egypt has reported lower IC_50_ than seen in our study. Although that study used the EO extracted from the same Mexican berries, the IC_50_s ranged between 3 and 12 *μ*g/mL when a panel of colon, hepatic, pulmonary, and intestinal cancer cell lines were treated [57]. This difference may be due to the use of the human macrophage cell line in our study or due to the Mexican berries gathered from different regional sources under different environmental conditions.

The RO EO cytotoxicity has also been reported in the literature. Interestingly, high IC_50_ > 250 *μ*g/mL was reported when the oil was exposed to a panel of ovarian and hepatic cancer cell lines [58], whereas a low IC_50_ of 8.5 *μ*g/mL, similar to our 14.15 *μ*g/mL, was calculated after exposure to pulmonary cancer cell line [36]. Again, all these discrepancies can be attributed to the composition of the EOs.

**Figure 2:**
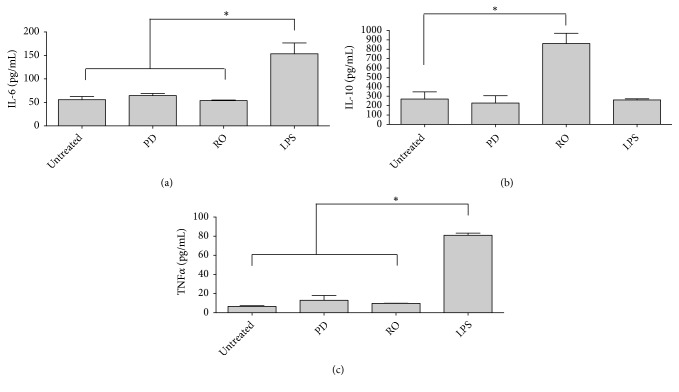
*Immunological response of EO*. The immunological response of the* Pimenta dioica* and* Rosmarinus officinalis* EOs was assessed on human-derived macrophage THP-1 cell line using ELISA for (a) IL-6, (b) IL-10, and (c) TNF-*α*. PD:* Pimenta dioica*. RO:* Rosmarinus officinalis*. LPS: lipopolysaccharide (positive control). Shown is the mean ± S.D. of three independent experiments. *∗*: *P*-value <0.05.

In the case of the anti-inflammatory activity, both EOs were not able to elicit a proinflammatory response because the levels of IL-6 and TNF-*α* were not significantly different from the untreated control (Figures 2(a) and 2(c)). Surprisingly, the levels of IL-10 (anti-inflammatory activity) of the RO EO showed a 4-fold increase compared to the untreated control (Figure 2(b)).

A previous study using ground extracts of PD reported an increase of 150% and 166% of the proinflammatory cytokines IL-6 and TNF-*α*, respectively [59]. The discrepancies with our studies are based on (1) the different source of the material used (plant extract versus EO in our study) and (2) the reported percentages of increase which were normalized to the cytotoxicity values (using MTT), which cannot be compared to our results expressed in pg/mL. Eugenol, the major component of the PD EO, has been shown to modulate the inflammatory response when macrophages and lung tissues were challenged with LPS [60, 61]. The inhibition of the inflammatory response was based on the inhibition of the IL-6 and TNF-*α* as a result of its interference in the activation of the transcription factor nuclear factor-*κ*B as measured in a murine model [61].

Regarding the anti-inflammatory activity of RO EO, a high concentration of the anti-inflammatory cytokine IL-10 was measured in our study, but an increase of the proinflammatory cytokines was not observed. Another study has reported a reduction of the IL-6 cytokine measured in the mice's colons [62], but no information was provided related to the amount of IL-10 secreted. Interestingly, another study reported a reduction of carrageenan-induced edema in a rat model, suggesting that the RO EO activates another anti-inflammatory pathway [63]. This fact is supported by other studies that showed that eucalyptol (one of the major compounds) in the RO EO reduced the inflammation in a carrageenan paw edema induced in mice and rats [18, 64]. Similarly, human gingival fibroblasts showed a decrease between 67 and 76% in the expression of IL-6 when exposed to eucalyptol and camphor, another compound identified in the EO [65]. Finally, a decrease in the level of TNF-*α* was measured when guinea pigs were challenged with ovalbumin and treated with eucalyptol [66]. These results are not surprising because eucalyptol and camphor are ingredients in over-the-counter medicines to treat coughs, such as VapoRub® and Buckley's®.

In our study, we found that the EOs show cytotoxicity when exposed to the cell line THP-1. It is clear that the addition of oils to the culture will have a direct contact with the cell membranes and always alter their composition, with detrimental effects to the viability of the cell. However,* in vivo* experiments showed different results. For example, a wound treatment of diabetic mice showed a better recovery when the animals were treated with the RO EO, compared to the aqueous extraction [16]. Also, an anti-inflammatory effect was observed when eucalyptol alone was used to treat patients with severe asthma [67]. Lastly, human lymphocytes and macrophages treated with eucalyptol showed a significant decrease in the secretion of proinflammatory cytokines [68].

## 4. Conclusions

The bioactivities of the EOs extracted from PD and RO were assessed. Results of these experiments showed that both EOs have antimicrobial activity and the RO EA was able to significantly increase the level of the anti-inflammatory cytokine IL-10. In summary, the novelty of this study is the antifungal activity of the EOs against the fungal pathogen* C. albicans* together with the absence of an inflammatory activity when EOs were exposed to macrophages. In addition, the RO EO showed a potent IL-10-dependent anti-inflammatory activity. Taken together, both oils can be used not only for topical applications as antimicrobials but also as anti-inflammatory agents. In addition, both oils can be used as antiseptics, such as in mouthwashes, topical creams or gels, or disinfectants.

## Figures and Tables

**Table 1:** Antimicrobial activity of PD, and RO EOs expressed as MIC (*μ*g/mL).

EO	Bacteria					Fungi			
	AB	EC	MRSA	PA	SA	AF	CA	CN	TR
PD	500	R	500	500	2000	R	600	R	R
RO	500	R	R	R	R	R	600	R	R
Control	0.1^ak^	10^g^	60^g^	10^ak^	1^g^	2^am^	2^am^	2^am^	1^tb^

AB, *Acinetobacter baumannii*; EC, *Escherichia coli*; MRSA, methicillin-resistant *Staphylococcus aureus*; PA, *Pseudomonas aeruginosa*; SA, *Staphylococcus aureus*; AF, *Aspergillus fumigatus*; CA, *Candida albicans*; CN, *Cryptococcus neoformans* var. *grubii*; TR, *Trichophyton rubrum*. R, resistant strain. PD, *Pimenta dioica* EO; RO, *Rosmarinus officinalis* EO. Ak, amikacin; Am, amphotericin; G, gentamicin; Tb, terbinafine.

## Data Availability

The data used to support the findings of this study are available from the corresponding author upon request.
